# Revision of Partulidae (Gastropoda, Stylommatophora) of Palau, with description of a new genus for an unusual ground-dwelling species

**DOI:** 10.3897/zookeys.614.8807

**Published:** 2016-09-01

**Authors:** John Slapcinsky, Fred Kraus

**Affiliations:** 1 Florida Museum of Natural History, University of Florida, Gainesville, FL 32611 USA; 2 Department of Ecology and Evolutionary Biology, University of Michigan, Ann Arbor, MI 48109 USA

**Keywords:** Land snail, Oceania, new species, Pacific, Palaopartula, Partula, pulmonate

## Abstract

We describe a new stylommatophoran land snail of the family Partulidae from Palau. The new species has a combination of morphological and ecological characters that do not allow its placement in any existing partulid genus, so we describe a new genus for it. The new genus is characterized by a large (18–23 mm) obese-pupoid shell; smooth protoconch; teleoconch with weak and inconsistent, progressively stronger, striae; last half of body whorl not extending beyond the penultimate whorl; widely expanded and reflexed peristome; relatively long penis, with longitudinal pilasters that fuse apically into a fleshy ridge that divides the main chamber from a small apical chamber; and vas deferens entering and penial-retractor muscle attaching at the apex of the penis. Unlike all other partulids, the new species is strictly associated with rocks in contact with the ground. Comparing the other three Palauan species – currently assigned to *Partula* – to our new genus and to other partulids makes it clear that they require their own genus because their morphology is quite different from that of true *Partula* and from that of all other genera. Hence, we resurrect the name *Palaopartula* Pilsbry for these snails.

## Introduction

The land-snail fauna of oceanic islands in the Pacific is disharmonic, with about 20 of the nearly 130 terrestrial snail families represented. Except for camaenid and bradybaenid species near the Asian and Australian continental margins (Chiba 2004, Hirano et al. 2014), helicoid families are noticeably lacking on Pacific Islands despite being the most diverse larger-bodied snails in adjacent continental areas. Oceanic islands instead are rich in operculate and smaller-bodied snails, presumably because these are more successful dispersers to far-flung islands (Cowie and Holland 2006). Nearly half of the families on oceanic islands in the Pacific – Assimineidae, Cyclophoridae, Diplommatinidae, Helicinidae, Hydrocenidae, Neocyclotidae, Pupinidae, and Truncatellidae – are operculates, of which there are only about 20 terrestrial families globally. The other families are eupulmonates that include moderate-sized Ellobiidae; moderate- to small-bodied Succineidae, Rhytididae, Charopidae, Endodontidae, Punctidae, Euconulidae, Trochomorphidae, and Zonitidae; and moderate- to small-bodied orthurethrans in the families Achatinellidae, Draparnaudiidae, Partulidae, Vertiginidae, and Amastridae (Cowie et al. 1995, Cowie and Robinson 2003, Rundell 2010). Orthurethrans, once thought to be ancestral stylommatophorans with relict island lineages, like Partulidae and Achatinellidae (Solem, 1990), are now viewed as a derived clade (Wade et al. 2006).

More than 50% of all extinctions documented since 1500 are mollusks; nearly 40% of these are terrestrial snails from oceanic islands (Régnier et al. 2009). Whereas the conservation status of most terrestrial vertebrate species has been assessed, only a small fraction of described species of terrestrial mollusks has been evaluated (Régnier et al. 2015a); as a result very few species are listed under protective legislation, even in groups clearly in decline (Régnier et al. 2015b). This lack of attention masks the extinction of many land snail species (Régnier et al. 2009, 2015a). Non-marine mollusks also suffer from poor sampling. Basic survey data for terrestrial mollusks are lacking for many Pacific Ocean islands, and many species face extinction before they are ever discovered (Richling and Bouchet 2013).

Palau harbors an estimated 200 species of land snails, of which only 78 have been described (Rundell 2010, Yamazaki et al. 2013). Ninety-five percent of the fauna discovered to date is endemic to these islands (Rundell 2010), and future discoveries are likely to prove so too. As currently known, the fauna comprises approximately 70% operculates and 30% eupulmonates, but of the 34 eupulmonates reported from Palau, five are alien (Rundell 2005). Among the 29 native eupulmonates, Helicarionoidea comprises the largest number of species, with 13; most of the others are tiny species of the families Achatinellidae, Charopidae, Endodontidae, and Succineidae. The helicarionoids, along with three partulids and one ellobiid (*Pythia scarabaeus* Linnaeus), include the only moderately large eupulmonates known from Palau. Hence, the land snails of Palau are, at the familial level, only a subset of the already disharmonic snail fauna that characterizes Pacific oceanic islands more generally.

During a visit in 1998, one of us (FK) discovered an unusual stylommatophoran snail of moderate size that was not readily assignable to any genus known from the country. Subsequently, we discovered that the species had been collected previously by others and that specimens were already lodged at the Florida Museum of Natural History. In trying to identify these specimens we confirmed that they were morphologically distinct from any known Pacific-island species. Herein we provide evidence for their familial assignment to Partulidae and describe a new genus and species for these specimens. In comparing this snail to its closest relatives it became obvious that Palauan snails currently assigned to *Partula* in fact do not accord well morphologically with that genus. Furthermore, they have been found to comprise a separate clade of partulids evolutionarily independent of lineages that are properly assigned to *Partula* (Lee et al. 2014). To facilitate comparisons with our new genus, we first resurrect *Palaopartula* Pilsbry for the three known species of Palauan partulids. We then describe our new genus and species.

## Materials and methods

**Figure 1.**
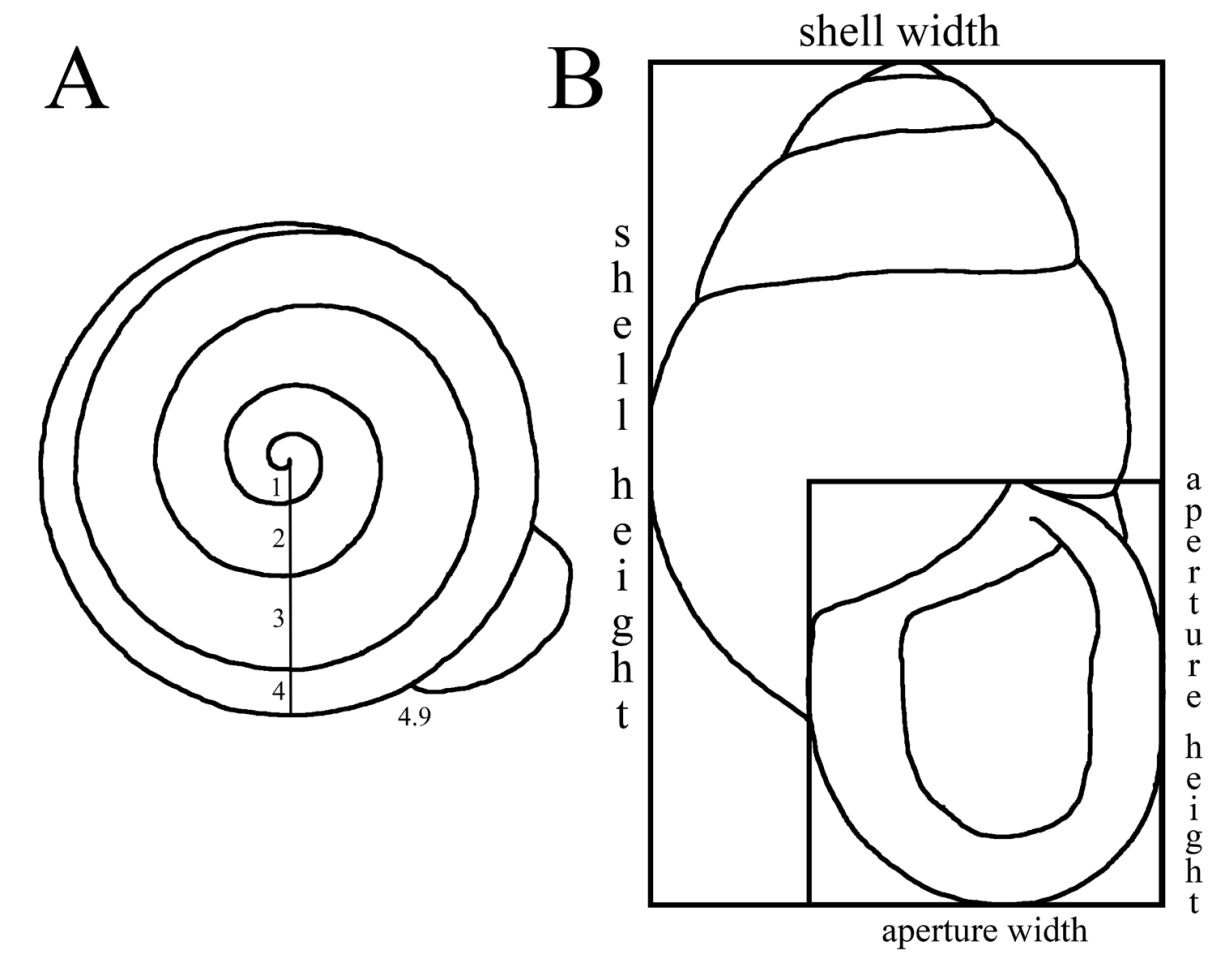
Shell measurements: **A** Whorl count **B** Shell width, shell height, aperture width, aperture height.

We hand-collected specimens, drowned them overnight, and preserved them in 75% ethanol. We dissected pallial organs, genitalia, and buccal masses from specimens under 75% ethanol using a dissecting microscope, and we isolated radulae from buccal masses using a 3% sodium hypochlorite solution. We imaged radulae and jaws using a Field Emission-SEM, photographed genital anatomy, and drew pallial organs with the aid of a drawing tube. We used Helcion Focus software for photo stacking. We counted whorls from the suture of the first whorl to the body whorl (Fig. 1A), and fractions of a whorl were determined with the aid of a cardboard circle divided into ten equal parts of 36°. We measured shells to 0.1 mm. Width is the greatest width of the shell perpendicular to the shell axis, and height is the greatest distance between the apex and the base of the aperture parallel to the shell axis (Fig. 1B). We include the expanded lip in both measures. Aperture width is the greatest distance from the columellar edge to the distal edge of the aperture, including the expanded lip. Aperture height is measured from the suture to the base of the expanded lip, parallel to the shell axis (Fig. 1B). We express measurements as means, ranges, and standard deviations. Specimens are deposited at the Florida Museum of Natural History
(UF). We also studied material from Academy of Natural Sciences, Philadelphia (ANSP) and from the Fred Kraus collection (FK), see Appendix 1. Higher-level systematics follows Bouchet and Rocroi (2005).

We isolated genomic DNA from 1 cubic mm of foot tissue of the new taxon using a solution of 10% Chelex beads (Bio-Rad Laboratories) in sterile water heated to 65 °C for 4 hours. DNAses and other protein contaminants were digested using Proteinase K. We amplified a 655-bp nucleotide fragment of cytochrome oxidase I (COI) with GoTaq DNA Polymerase (Promega, Madison, WI) using the primer pairs LCO1490/HCO2198 (Folmer et al. 1994) and were able to sequence 540 bp of that. Sequencing was performed in both directions at University of Florida, Interdisciplinary Center for Biotechnology Research, using their standard procedures. The sequence is deposited in GenBank under accession number KX685957. Comparative COI sequences were provided by the authors of Lee et al. (2014). We conducted a phylogenetic analysis of our taxon versus other partulids using Bayesian analysis in MrBayes (Ronquist and Huelsenbeck 2003) as implemented in Geneious version 6.1.7 (http://www.geneious.com, Kearse et al. 2012). The GTR + I + G model of evolution was selected using jModelTest 2.0 (Darriba et al. 2012). We ran the analysis for 10,000,000 generations running four chains: one cold and three heated to a temperature coefficient of 0.2. Trees were sampled every 10,000 cycles after a burn-in period of 100,000 generations. We present our analysis unrooted because other orthurethran outgroups available to us for rooting are distantly related to partulids, we could not obtain a stable root using them, and they are likely uninformative due to their long branch lengths.

### Familial assignment to Partulidae

**Figure 2.**
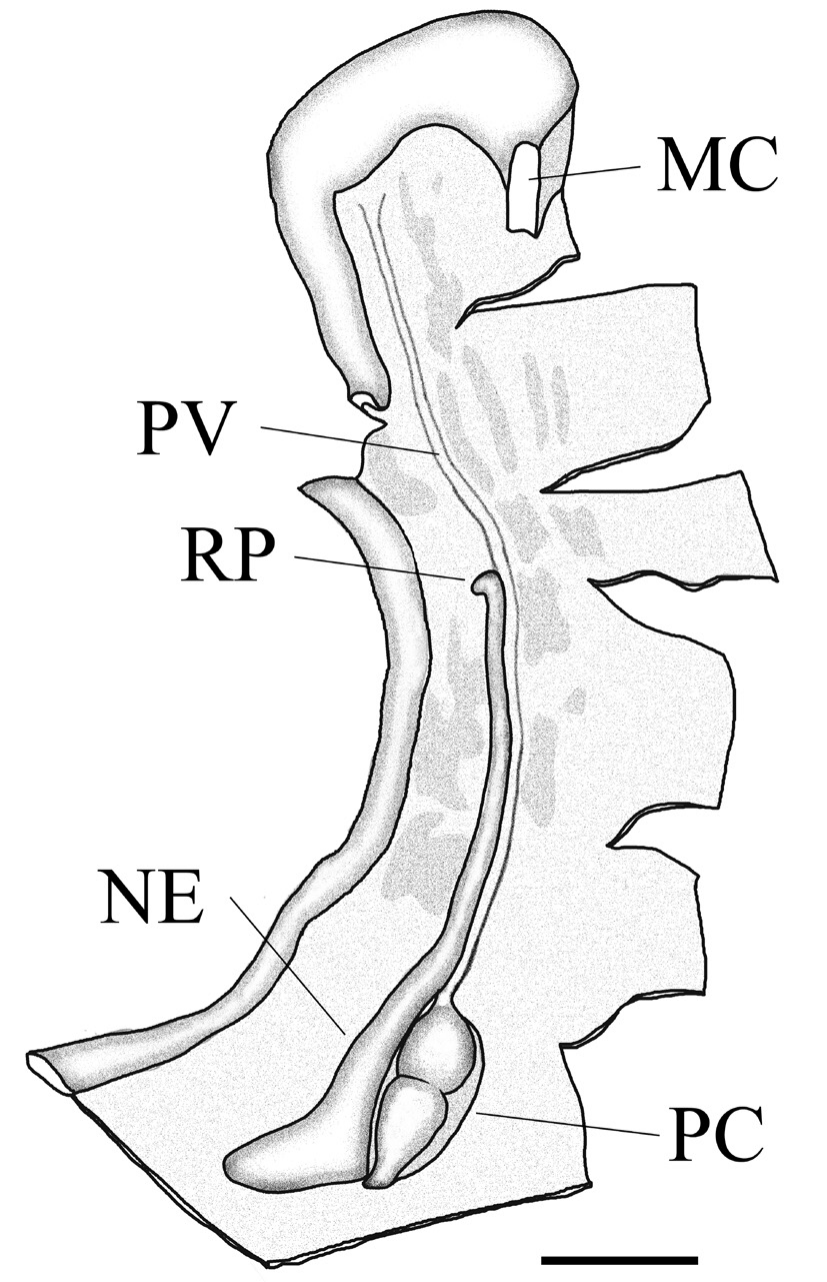
Pallial organs of *Sphendone insolita* sp. n., paratype, UF 248690. MC = mantle collar; NE = nephridium; PC = pericardium; PV = pulmonary vein; and RP = renal pore. Scale bar: 5 mm.

**Figure 3.**
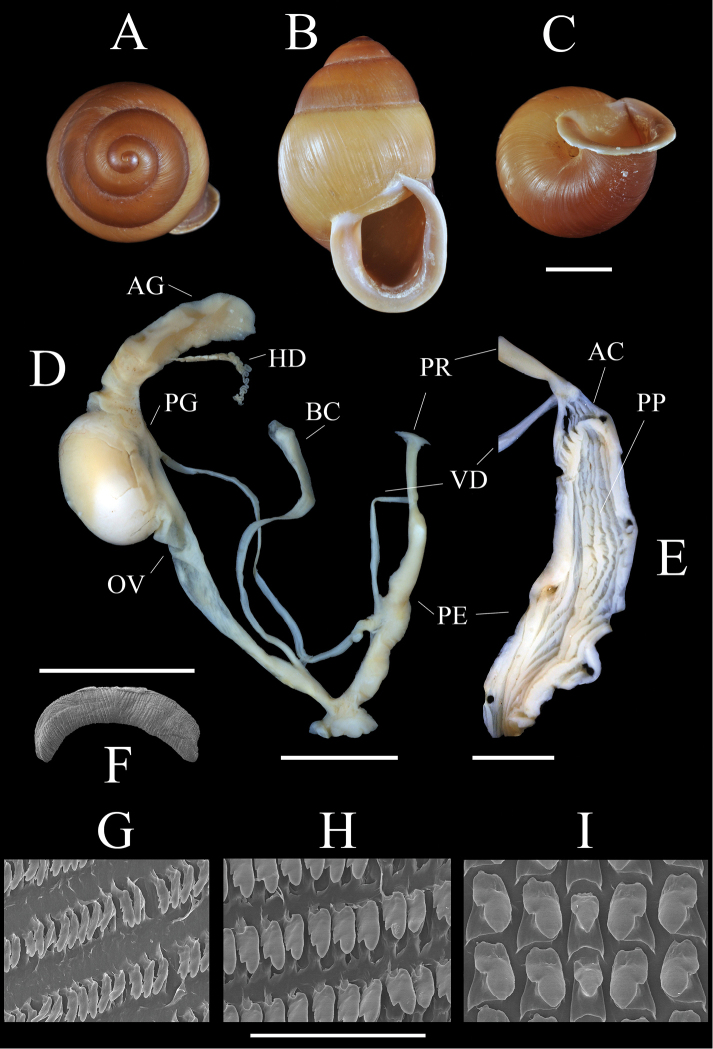
*Sphendone insolita* sp. n.: **A–C** Shell, holotype, UF 425857 **D** Genitalia, paratype, UF 248690 **E** Penial sculpture, paratype, UF 248690 **F** Jaw, paratype, UF 248690 **G–I** Radula, holotype UF 425857. AC = apical chamber; AG = albumen gland; BC = bursa copulatrix; HD = hermaphroditic duct; OV = free oviduct; PE = penis; PG = prostate gland; PP = penial pilasters; PR = penial retractor muscle; and VD = vas deferens. Scale bar: 5 mm (**A–C, D**); 2 mm (**E**); 1 mm (**F**); 100 µm (**G–I**).

The familial placement of this new, unusual stylommatophoran species is not immediately obvious based on shell characters. The shell resembles some Camaenidae and Bradybaenidae, but these are precluded because the new species lacks a head wart, which is present in many camaenids (Scott 1998), and the pallial complex is orthurethran (Fig. 2) instead of sigmurethran, as in both Bradybaenidae and Camaenidae. Of the orthurethran families, the large pupoid shell with greatly expanded lip (Fig. 3A–C) is most closely approached by the Achatinellidae, Draparnaudiidae, Partulidae, and Enidae. The shells are much larger (18–23 mm) and lack the apertural lamellae of the pupilloid families. The radula has three tooth types (Fig. 3G–I) and differs from that of the Achatinellidae, which has only a single tooth type, considered marginal by Cooke and Kondo (1960). The pallial organs (Fig. 2) are not pseudosigmurethran, as in the Cerastidae (Solem 1964; Budha et al. 2012). The shell does not have a columellar lamella, like the Amastridae and many Cochlicopidae. The widely expanded lip is unlike the simple or thinly reflected or thickened lips of the Achatinellidae, Amastridae, Cerastidae, and Cochlicopidae but is typical of some Enidae and the partuloid families Partulidae and Draparnaudiidae. The Enidae and Draparnaudiidae differ by having penial accessories (Tillier and Mordan 1995, Schileyko 1998) that are absent in this species. Among stylommatophorans found in the Pacific region that are >10 mm in maximum dimension, all morphological features examined by us are consistent only with the Partulidae. For these reasons we assign this new genus and species to that family. Biogeographically, this is reasonable inasmuch as the family is already known from Palau and adjacent areas, whereas several of these other orthurethran families are not. Further, a BLAST comparison of a 540-bp fragment of COI recovered from the new species (UF 271885) confirmed the sequence was most similar to sequences of other partulid species in GenBank (see below).

### Notes on terminology of genital anatomy in Partulidae

The penis in *Samoana*, *Palaopartula* and *Partula* is divided into two chambers: a main chamber and an apical chamber that are usually defined by a constriction between them and by different sculpture. Pilsbry and Cooke (1934) considered the apical chamber in *Samoana* to be an epiphallus, which is a specialization of the vas deferens, whereas they referred to the distal or apical chamber in *Partula* and *Palaopartula* as a specialization of the penis. However, they also suggested that the epiphallus in *Samoana* and the distal chamber of the penis in *Partula* may be homologous structures, and they urged further research to determine function and settle on a fixed terminology for these structures in various partulid genera (Pilsbry and Cooke 1934). Schileyko (1999) referred to the apical chamber in *Partula* and *Palaopartula* as a flagellum, which is a specialization of the epiphallus, and this conflicts with Pilsbry and Cooke’s interpretation of this structure as a specialization of the penis. Schileyko’s interpretation suggests that the apical chamber (epiphallus) of *Samoana*, *Partula* and *Palaopartula* are homologous. Although we agree that these structures are likely to be homologous, we prefer to use the term “apical chamber” rather than “epiphallar chamber” for this structure because it does not imply knowledge of its function or origin. We employ the terminology of Pilsbry and Cooke (1934) for the apical chamber in *Samoana* and Schileyko (1999) for the apical chamber in *Partula* and *Palaopartula* in Figure 6 to aid comparisons.

## Systematic descriptions

### Family Partulidae Pilsbry, 1900

#### Genus Palaopartula

Taxon classificationAnimaliaStylommatophoraPartulidae

Pilsbry, 1909

Palaopartula Pilsbry, 1909, in Pilsbry 1909–1910, pages 166, 306.
Palaeopartula Richardson, 1990, page 6 [incorrect subsequent spelling].
Palaopartula Schileyko, 1999, page 271, figure 327.

##### Type species.

*Partula thetis* Semper, 1865; by original designation.

##### Content.

*Partula calypso*, *Partula leucothoe*, and *Partula thetis*, all Semper 1865.

##### Distribution.

Known only from Palau.

##### Diagnosis.

**Figure 4.**
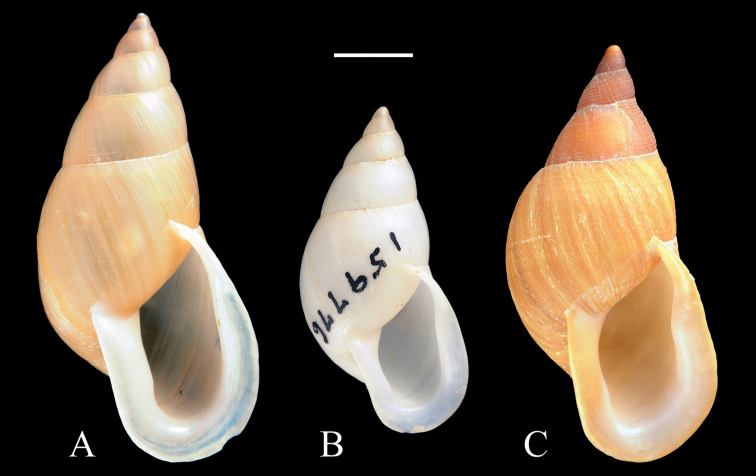
Shells of *Palaopartula*: **A** *Palaopartula calypso*, ANSP 191976 **B** *Palaopartula leucothoe*, ANSP 294471 **C** *Palaopartula thetis*, FK 2840. Scale bar: 5 mm.

Shell large, elongate, with a tall, relatively flat-sided and acutely pointed spire (Fig. 4). Protoconch small, early teleoconch whorls descend rapidly, remaining tightly coiled and narrow. Aperture elongate, with a greatly expanded peristome, its palatal and parietal sides nearly parallel, palatal edge with a slight central thickening, base rounded, parietal edge joining high on the body whorl. Umbilicus very deep and not covered by the parietal callus. Penis long and narrow, divided nearly equally between a main chamber with strong pilasters running its length that are crossed by weaker pilasters and an apical chamber with narrow pilasters or pustules (Kondo 1955, figs 118–123).

##### Comparisons with other genera.

**Figure 5.**
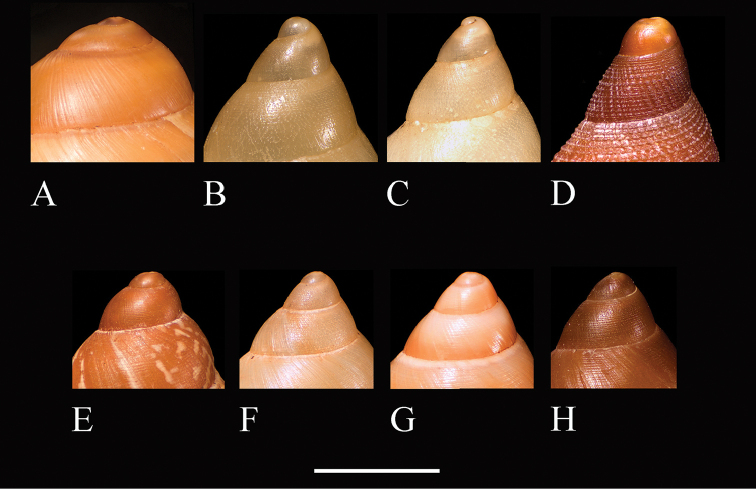
Apical sculpture of partulid genera: **A** *Sphendone insolita* sp. n., holotype, UF 425857 **B** *Palaopartula calypso*, ANSP 191976 **C** *Palaopartula leucothoe*, ANSP 294471 **D** *Palaopartula thetis*, FK 2840 **E** *Eua zebrina* (Gould, 1847), UF 158688 **F** *Samoana strigata* (Reeve, 1850), UF 192725 **G** *Partula gibba* Ferussac, 1821, UF 195878 **H** *Partula varia* Broderip, 1832, UF 158682. Scale bar: 5 mm except 2.5 mm for *Palaopartula* species.

**Figure 6.**
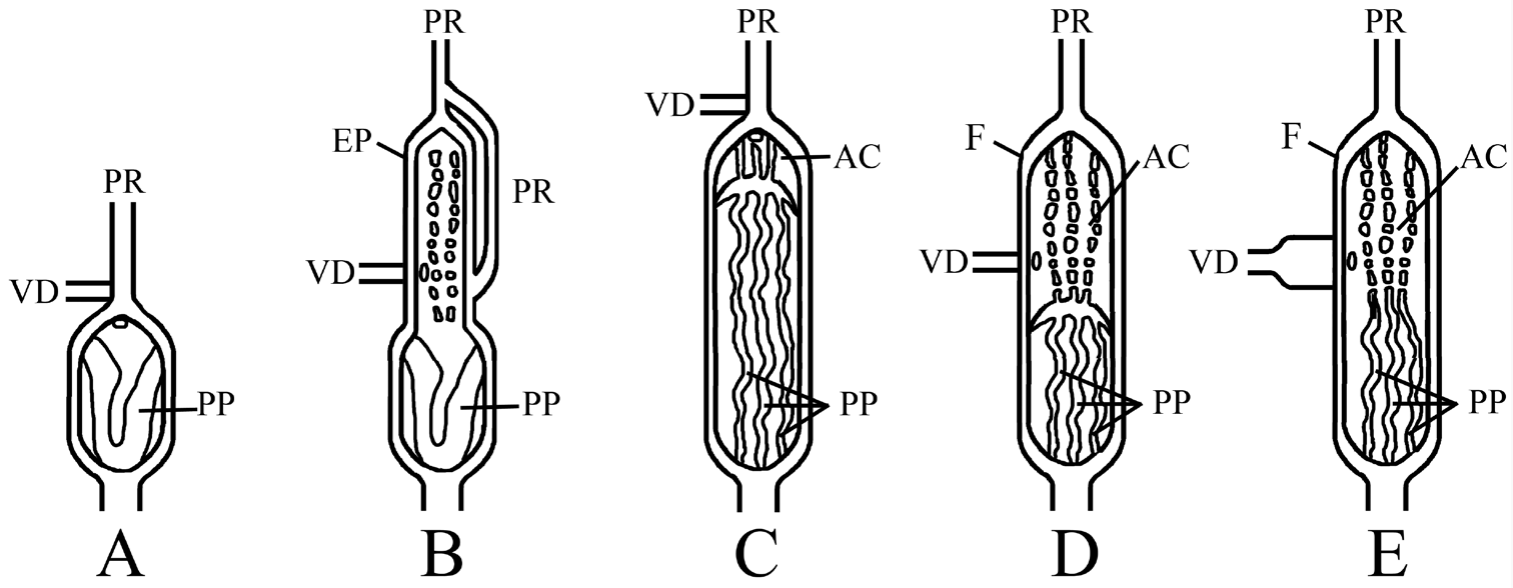
Schematic summary of penial anatomy of partulid genera, from Kondo (1955) and Schileyko (1999): **A** *Eua* **B** *Samoana* **C** *Sphendone* **D** *Palaopartula* **E** *Partula*. AC = apical chamber; EP = epiphallus; F = flagellum, PP = penial pilasters; PR = penial retractor muscle; and VD = vas deferens.

*Palaopartula* has historically been grouped with *Partula*. It differs from *Partula*, *Eua*, and *Samoana* in having a relatively large and more elongate shell with a high and rapidly descending spire (Fig. 5B–D). In contrast, *Partula* (Fig. 5G–H), *Eua* (Fig. 5E), and *Samoana* (Fig. 5F) have relatively blunt apices, with rounded whorls that expand relatively rapidly and descend slowly. The height/width ratio range of the three *Palaopartula* species is 2.0–2.3, versus 1.5–1.9 for the *Partula*, *Eua* and *Samoana* species listed in Appendix 1. The early teleoconch whorls of *Palaopartula* are variable in sculpture: *Palaopartula calypso* (Fig. 5B) and *Palaopartula leucothoe* (Fig. 5C) have pitted spiral striae like most *Partula* and *Samoana*, although the spiral striae are much weaker; *Palaopartula thetis* (Fig. 5D) is unlike any other partulid in being sculptured with raised axial and spiral sculpture that join to form nodules. The peristome of *Palaopartula* species is more widely reflected than in any other genus of Partulidae, and the parietal edge attaches to the shell higher on the body whorl and does not obstruct the umbilicus, making the umbilicus look particularly deep. *Palaopartula* differs from *Eua* in having a long thin penis (Fig. 6D), unlike the short broad penis of *Eua* (Fig. 6A). Internally the penis of *Palaopartula* is divided into two chambers, a main penial chamber and an apical chamber, each with 5–15 longitudinal pilasters; this contrasts with *Eua*, which has only one chamber with one large fleshy pilaster (Kondo 1955). The vas deferens of *Palaopartula* joins the apical chamber laterally rather than joining the penial chamber apically, as in *Eua*. The penial and apical chambers of *Palaopartula* are similar in width and length and both contain 5–15 longitudinal pilasters, whereas the penial chamber of *Samoana* is short and bulbous with only two large pilasters that fuse to form a V (Fig. 6B), and the apical chamber is long and narrow and contains 5–10 longitudinal rows of nodules. *Palaopartula* has an unbranched penial retractor muscle that attaches apically as opposed to the retractor muscle of *Samoana* which also has a secondary branch that attaches to the penial chamber (Fig. 6B). *Palaopartula* differs from *Partula* in having a narrow and usually tapering penis, whereas *Partula* has a more variable penis that is usually apically inflated and strongly curved (Kondo 1955). In *Palaopartula*, the pilasters in both chambers fuse to form a ridge between the chambers, unlike *Partula*, which has pilasters that do not fuse to form a ridge between the two chambers (Fig. 6E). The vas deferens of *Palaopartula* remains narrow for its entire length whereas the vas deferens of *Partula* broadens before entering the apical chamber. Molecular data also do not support placement of *Palaopartula* within *Partula* (Lee et al. 2014) but link it instead with *Samoana*, from which it differs radically in shell and genital morphology. Accordingly, we here resurrect *Palaopartula*.

##### Remarks.

**Figure 7.**
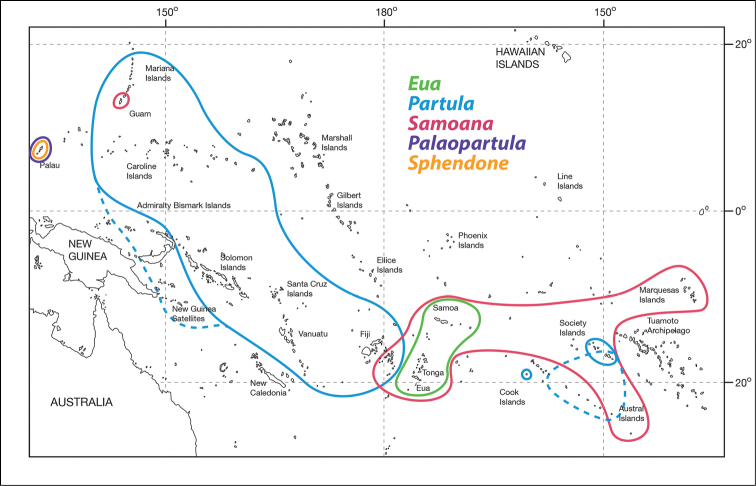
Geographic ranges of the five partulid genera. Map modified from Lee et al. (2014).

**Figure 8.**
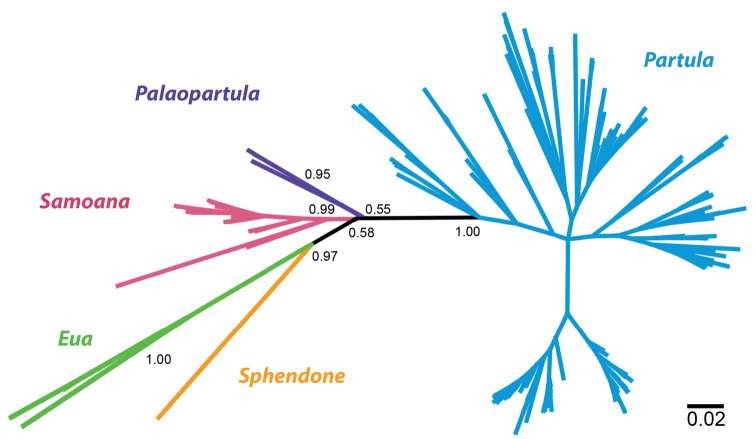
An unrooted network of partial COI sequences (540 bp for *Sphendone*, 655 bp for the other species) of Partulidae constructed using MrBayes as implemented in Geneious version 6.1.7. Node labels are posterior probabilities. The scale bar is equal to 2% sequence divergence.

This genus is isolated to the west of all previously named partulid genera (Fig. 7). The species are arboreal, with *Palaopartula thetis* typically being found in *Pandanus* leaf axils (FK, pers. obs.). Judging by genetic distances, *Palaopartula* is distantly related to *Partula* but clusters more closely to the other partulid genera (Fig. 8), although we are unable to polarize this network to determine directionality of evolution.

#### Genus Sphendone gen. n.

Taxon classificationAnimaliaStylommatophoraPartulidae

http://zoobank.org/FBC28415-B5A9-4549-B4CC-27D0F7B9850B

##### Type species.

*Sphendone insolita* sp. n.

##### Content.

One species, *Sphendone insolita* sp. n.

##### Diagnosis.

Shell obese-pupoid (Fig. 3B), protoconch sculptured with growth lines, strongest near the apical suture (Fig. 5A); spiral sculpture absent or consisting of very weak and inconsistent striae. Wavy spiral striae become progressively stronger and clearer on the penultimate and body whorls. The final half of the body whorl does not extend beyond the penultimate whorl (Fig. 3A–B). Peristome widely expanded and reflexed, palatal and parietal margins nearly parallel, base evenly rounded. Palatal margin with slight central thickening; parietal margin attaching to body whorl at level of umbilicus. Penis relatively long and narrow, sculptured with approximately 10 longitudinal pilasters that coalesce to form a ridge between the penis and a very short and thin-walled apical chamber with about 10 thin and widely spaced pilasters. The vas deferens and penial-retractor muscle enter at the penis apex. Oviduct usually contains only one (sometimes two) eggs or embryos.

##### Comparisons with other genera.

The shells of *Eua*, *Samoana*, *Palaopartula*, and *Partula* are not pupoid in shape; they are attenuated apically. The protoconch of the new species lacks strong spiral striae or pitting, unlike the strong striae of *Eua*, or the pitted striae of *Palaopartula*, *Partula*, and *Samoana*. *Sphendone* is similar only to *Eua* in the apical insertion of the vas deferens into the penis, unlike the lateral insertion into the apical chamber in *Samoana*, *Palaopartula*, and *Partula* (Figure 6). However, *Sphendone* shares an elongate, two-chambered penis with *Samoana*, *Palaopartula*, and *Partula* that is distinctly different from the short, broad penis with a single chamber found in *Eua*. The apical chamber of *Sphendone* is very short, unlike *Samoana*, *Palaopartula*, and *Partula*, which have large apical chambers (Fig. 6). The interior of the penis in *Sphendone* is sculptured with approximately ten longitudinal pilasters, unlike the single fleshy pilaster of *Eua* or the two large pilasters in the main chamber of the penis of *Samoana* (Kondo 1955). The pilasters of the main and apical chambers coalesce, forming a ridge between the two chambers similar to that seen in *Palaopartula* and unlike that in *Samoana* and *Partula*. The penial-retractor muscle enters apically and does not branch, whereas *Samoana* has a retractor muscle with a secondary branch that attaches to the penial chamber.

##### Remarks.

A comparison of a partial COI sequence (540 bp) of *Sphendone* (UF 271885) with sequences from all other partulid genera (655 bp) shows the new genus is well differentiated from other partulid genera (Fig. 8) although we are unable to determine its sister-taxon relationships from an unrooted network.

##### Etymology.

*Sphendone* is a feminine Greek noun for a sling missile and is used here in reference to the unique bullet shape of the shell among partulids. The accent is on the first syllable.

##### Distribution.

Known only from Palau.

#### Sphendone insolita sp. n.

Taxon classificationAnimaliaStylommatophoraPartulidae

http://zoobank.org/2CC94994-C317-4CDA-A815-D07C1084FD8C

Figs 2 , 3

##### Holotype.

UF 425857, 7.2600°N, 134.4493°W, collected along trail to German Lighthouse, Ngeruktabel (Uruktapel) Island, Palau, F. Kraus, 24 August 1998.

##### Paratypes

**(n = 76).** Palau: Ngeruktabel (Uruktapel) Island: along trail to German Lighthouse, 7.2600°N, 134.4493°W, F. Kraus, 24 August 1998 (UF 271885, 8 alcohol preserved, UF 271886, 16 dry shells); at ruins of Japanese artillery battery, 150 meters, F. G. Thompson, 23 October 1985 (UF 248690, 16 alcohol preserved; UF 248689, 31 dry shells); along mossy wall downhill of compound ruins, J. Starmer, 21 August 1999 (UF 332693, 6 dry shells).

##### Other material examined.

Palau: Eil Malk (Mecherchar) Island: southeastern peninsula, from hermit crab, F. G. Thompson, 22 October 1985 (UF 249044, 1 dry shell).

##### Diagnosis.

Large, obese-pupoid shell approximately 18–23 mm in height and 13–15 mm in width, with 4.5–5.1 whorls. Body whorl descends below the penultimate whorl. Protoconch sculptured with growth lines, strongest near the sutures; spiral sculpture lacking or, less commonly, comprising faint and irregular striae. Wavy spiral striae are progressively stronger and more regular on penultimate and body whorls but never become regular and strong. Peristome widely expanded and reflexed, palatal and parietal margins nearly parallel, base evenly rounded. Palatal margin with slight central thickening; parietal margin attaching to the body whorl at umbilicus. Penis long, with apical insertion of the vas deferens and retractor muscle. Approximately ten longitudinal pilasters coalesce to form a ridge that defines a small apical chamber below the insertion of the vas deferens.

##### Description of holotype.

Shell obese-pupoid, height 22.2 mm, width 14.0 mm, with 4.9 whorls (Fig. 3A–C). Whorls moderately inflated, with impressed sutures. Apex somewhat bluntly rounded (Fig. 5A). Protoconch and teleoconch whorls sculptured with strong growth lines, especially near the sutures; there are also irregularly expressed, weak and wavy spiral striae that become progressively stronger in later whorls. Last whorl descending underneath the penultimate whorl. Aperture elongate, sides nearly straight and parallel. Outer edge of peristome curving slightly inward at middle, base evenly rounded. Aperture height 12.1 mm, width 9.3 mm. Interior of aperture brown. Peristome nearly complete, broadly expanded and reflexed, thickened, brown towards the aperture, fading to brown-white and reflexed abaperturally. Parietal callus thin in the middle and much thickened at the inner and outer insertions of the lip. Shell color medium brown, with a poorly defined paler band below the suture on early whorls that widens on subsequent whorls. Penultimate whorl pale brown to straw yellow-brown except for a poorly defined and narrow darker-brown band below the suture. Body whorl darkens, the second half entirely darker brown.

**Variation.** Measurements of 56 adult shells: height 18.3–22.7 mm, mean 21.1 ± 0.8 mm; width 12.9–14.9 mm, mean 14.0 ± 0.4 mm; whorls 4.5–5.1, mean 4.9 ± 0.1. Aperture height 10.5–12.1 mm, mean 11.3 ± 0.4 mm. Aperture width 8.4–9.8 mm, mean 9.2 ± 0.3 mm. Most shells are similar to the holotype in coloration, but three of 79 shells are uniformly white. These white-shelled individuals have normal surface sculpture and are not worn. Nor are they albino, as evidenced by their normal mantle pigmentation. Most shells are dextral, but two of 79 are sinistral.

**Pallial system (2 specimens).** Nephridium nearly 2/3 length of pallial cavity, broad at base, tapering anteriorly, and sharply turned at renal orifice (Fig. 2). Pericardium approximately 1/3 length of nephridium. Pulmonary vein long and unbranched, ending near mantle collar. Mantle lacks any other obvious venation.

**Radula (2 specimens).** Central tooth two-thirds height of lateral teeth, trigonal, with poorly defined ectocones (Fig. 3I, central row). First lateral teeth bicuspid, ectocone 1/3–1/2 height of broad mesocone, which narrows abruptly at the tip (Fig. 3I, flanking central row). First ten lateral teeth similar in size and shape. Next five rapidly becoming narrower and beginning to develop multiple ectocones (Fig. 3H). Next 80 teeth narrow, slowly grading into marginals, width approximately one-quarter height, with two to several ectocones (Fig. 3G).

**Jaw (1 specimen).** Crescent-shaped, thin, stegognathous, composed of many narrow flat plaits that converge towards middle of cutting edge, which is not raised and does not bear a central cusp (Fig. 3F).

**Reproductive system (10 specimens).** Prostate gland short, extending only a short distance beyond albumen gland (Fig. 3D). Vas deferens weakly attached by fibers above penial-oviduct juncture, attached strongly to penis at its mid-point and again to penial-retractor muscle just above insertion on apex of penis; entering penis apically. Penis relatively long, cylindrical, divided into two chambers; the main chamber sculptured with numerous longitudinal pilasters crossed perpendicularly by weaker pilasters; the apical chamber sculptured with relatively weak pilasters. Pilasters fuse at the junction of the two chambers, forming a ridge below the insertion of the vas deferens (Fig. 3E). Atrium very short. Vagina short. Bursa copulatrix receptical oblong, tapering gently and imperceptibly to its duct. Single large egg 6.4–6.6 mm, mean 6.5 ± 0.1 mm (n = 6), with hard calcareous shell (Fig. 3D) found in six of ten dissections, one of these also had an embryo; another embryo found in an individual without an egg. Hermaphroditic duct narrow and highly convoluted.

##### Comparisons with other species.

The new species differs from other partulids as stated for the genus.

##### Etymology.

The trivial name is a feminine Latin adjective meaning unusual, in recognition of both the unusual shell shape and ecological habits for a partulid.

##### Distribution.

**Figure 9.**
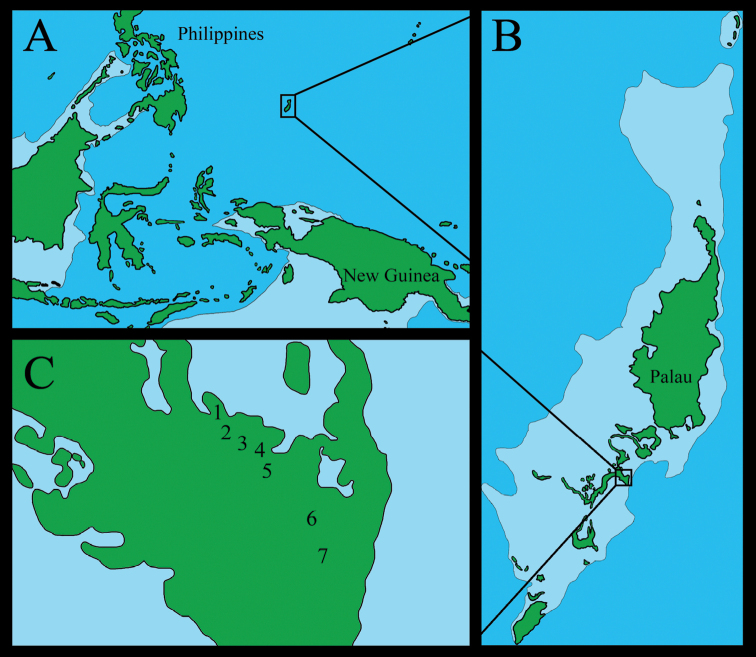
**A–C** GPS stations on Ngeruktabel Island, Palau, demarcating the trail to the German lighthouse, along which the type series of *Sphendone insolita* was collected.

Known only from southeasternmost Ngeruktabel (Uruktapel) and nearby Mecherchar (Eil Malk) Islands, Palau, Caroline Islands (Figs 7, 9). These two islands are separated by shallow waters and were connected in the past few thousand years, as the maximum depth between the two islands is only 25 m (Defense Mapping Agency 1996). Because all the hundreds of islands within the central fringing reef of Palau comprised a single island during the last glaciation event (Colin 2009), the species may be more widely distributed among other of the Rock Islands than is currently apparent.

##### Ecological notes.

**Figure 10.**
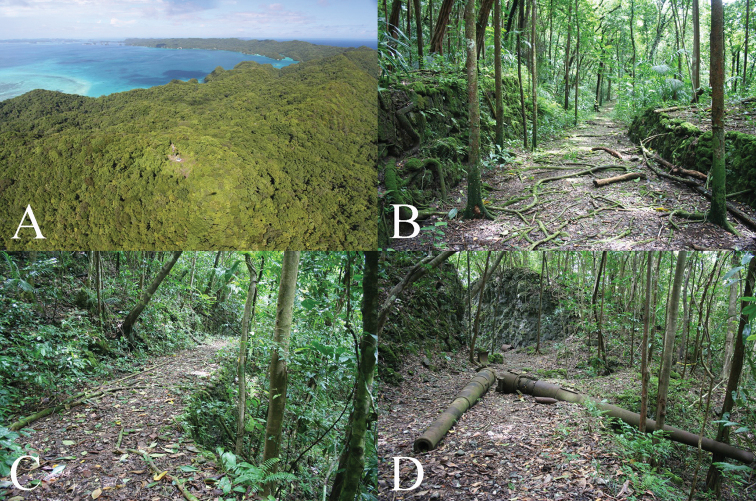
Photos of forested habitat at the type locality of *Sphendone insolita* on eastern Ngeruktabel Island. **A** Aerial view of the area, looking toward the southwest, showing the German lighthouse at the highest point **B–D** Forested trail along which the type series of *Sphendone insolita* was collected.

Live individuals of the new species were collected only from beneath rocks, between soil and the overlying rock, or deep in rock piles. Dead shells were also found on the soil surface and in leaf litter among stones. The site is well-developed limestone rainforest (Fig. 10) that was partially cleared during WWII but is now difficult to distinguish from undisturbed forest (Crombie and Pregill 1999).

## Discussion

Palau comprises more than 700 small islands at the western end of the Pacific Ocean, totaling 490 km^2^ of land and extending over 700 km, although the majority of islands lie within a single fringing reef approximately 130 km in length. These islands are situated some 800 km east of the Philippines and 850 km north of New Guinea and form the westernmost component of the Caroline Islands. They lie along the southern end of the Kyushu-Palau Ridge, a relict intra-oceanic volcanic-arc system the evolution of which ended 15–25 MYA (Yan and Shi 2011). Clockwise rotation of the Philippine Sea Plate brought the ridge to lie along the boundary of the Caroline Plate (Hall et al. 1995; Hawkins and Ishizuka 2009), and subsequent uplift left the islands as the only subaerial portion of the Kyushu-Palau Ridge (Neall and Trewick 2008). The islands consist of oceanic volcanics, many of which are capped with uplifted reef limestone (Hawkins and Ishizuka 2009), indicating prior submergence. During the last glaciation event, when sea levels were 120 m lower than today, all the hundreds of islands within the central fringing reef comprised a single island (Colin 2009). The islands of Palau have never been connected to any continental landmass (Hall et al. 1995; Hawkins and Ishizuka 2009), and their biotic colonization, like that of all oceanic islands, necessarily involved over-water dispersal (Cowie and Holland 2006). The broad distribution of Partulidae across the vast expanse of oceanic Pacific islands makes clear their exceptional dispersal abilities, so their presence among the largest native land snails of Palau is unsurprising.

Partulidae comprises more than 120 currently recognized species (Kondo 1955, 1968, Cowie 1992) endemic to the western and south-central Pacific from the Mariana Islands and Palau east to the Austral and Marquesas Islands (Fig. 7). The family is particularly diverse in the Society Islands, where they are among the largest and best-studied land snails (Crampton 1916, 1932, Murray and Clarke 1980, Cowie 1992, Lee et al. 2014). However, many species have declined or are extinct as a result of the loss of native lowland forest and human introduction of alien species, especially predators like *Euglandina rosea*, released in disastrous and unsuccessful biocontrol efforts (Murray et al. 1988). These losses have prompted zoo and lab-rearing efforts (Pearce-Kelly et al. 1997) followed by reintroduction to exclosures to prevent additional extinctions (Coote et al. 2004), and back into natural habitats in the wild (Coote et al. 2016). Relative to the species of *Palaopartula*, which appear to be generally rare (O’Foighil and Rundell 2012a, b, c), we found *Sphendone insolita* to be relatively common. Invasive rodents have been suggested as one agent for decline of *Palaopartula* species (O’Foighil and Rundell 2012a, b, c), and it may be that the fossorial habits of *Sphendone insolita* confer some degree of protection from those predators and allow for its larger numbers.

All known partulids, like many other Central Pacific taxa, including Microcystinae and some Achatinellidae, are ovoviviparous. Although ovoviviparous species may have an advantage colonizing islands (Tompa 1984) they often have low fecundity (Baur 1994). *Partula suturalis* reared under laboratory conditions average 353 days to reach adult shell size and an additional 192 days before producing their first offspring. Afterwards, a single offspring is born approximately every 22 days (Murray and Clarke 1966). Partulids produce fewer than 90 offspring during their approximately 5 year life-span, far fewer than other snails with similar life-spans (Cowie 1992). The oviducts of most adult *Partula* simultaneously contain both eggs and embryos. Gravid individuals of ten species inhabiting Moorea averaged 1.37 eggs and 1.13 embryos (Crampton 1932), and four species from the Mariana Islands averaged 1.94 eggs and 1.20 embryos (Crampton 1925). Seven gravid individuals of *Sphendone insolita* averaged only 0.85 eggs and 0.28 embryos, and their eggs are particularly large, averaging 6.5 mm in greatest width, or nearly 0.29 of the total shell height. This is relatively larger than *Partula solitaria*, which was noted for having particularly large eggs at 3.5 mm, or 0.22 of shell height (Crampton 1932). The relatively small number of large eggs and embryos in *Sphendone insolita* suggests this species reproduces relatively slowly compared even to other partulid species. Low reproductive rate is likely to make the new species particularly vulnerable to population suppression from introduced predators or habitat disturbance. In fact, *Partula* species on Tahiti that were rare but had the largest instantaneous clutch sizes (Crampton 1916) persist, whereas their more common congeners with smaller instantaneous clutch sizes have not (Bick et al. 2015).

*Sphendone insolita* is relatively common where found but may have specific habitat requirements that give it a limited and/or discontinuous distribution within Palau. Nearly all partulids are arboreal; however, a few species live on the ground in leaf litter – such as *Palaopartula compressa* and *Palaopartula crassilabris* – or in leaf litter and under stones – like *Palaopartula producta* (Pilsbry 1910). The new species is the only one associated with deep rock and boulder talus or found in crevices under and between rocks on the ground but not in surrounding leaf litter. Until further surveys better delimit this species’ range, populations should be considered spatially restricted, making this species potentially vulnerable to habitat modification such as deforestation or limestone mining. The habitat specificity, small range, and apparently slow rate of reproduction of this species should make this a species of special concern to land managers.

## Supplementary Material

XML Treatment for Palaopartula

XML Treatment for Sphendone

XML Treatment for Sphendone insolita

## Appendix 1

### Specimens examined

*Eua expansa* (Pease 1872): Samoa: Savaii Island, near Salailua, L. Price, November 1965 (UF 485789, 3 shells).

*Eua zebrina* (Gould 1846): American Samoa: Tutuila Island (UF 158688, 22 shells), C. M. Dumbauld, March 1941 (UF 29715, 3 shells).

*Palaopartula calypso* Semper 1865: Palau: Babeldaob Island, Oikull, 7.4122°N, 134.5833°E, F. G. Thompson, 17 October 1985 (UF 248936, 1 shell); Koror Island, small ridge, 7.3444°N, 134.5001°E, F. G. Thompson, 13 October 1985 (UF 248833, 2 shells); South Babelthaup Island, D. Thaanum (ANSP 191976, 18 shells).

*Palaopartula leucothoe* Semper 1865: Palau (ANSP 294471, 5 shells).

*Palaopartula thetis* Semper 1865: Palau: Ngermalk Island, 7.3351°N, 134.4583°E, F. G. Thompson, 10 October 1985 (UF 248593, 1 shell); Ngeruktabel Island: along trail to German Lighthouse, 7.2600°N, 134.4493°W, F. Kraus, 24 August 1998 (FK 2840, 4 shells); at ruins of Japanese artillery battery, F. G. Thompson, 23 October 1985 (UF 248688, 3 shells); northeast side of island, T. M. Iliffe, 11 February 1985 (UF 265851, 5 shells); South Babelthaup Island, D. Thaanum (ANSP 191960, 13 shells); Ulong Islands: west island, northwest shore, R. I. Crombie, 16 January 1995 (UF 252891, 9 shells).

*Partula affinis* Pease 1868: French Polynesia: Society Islands: Tahiti Island, Faarumai, W. H. Pease (UF 192743, 30 shells).

*Partula arguta* Pease 1864: French Polynesia: Society Islands: Huahine Island, A. Garrett (UF 111866, 6 shells).

*Partula auriculata* Broderip 1832: French Polynesia: Society Islands: Raiatea Island, A. Garrett (UF 490635, 27 shells); W. J. Clench (UF 192745, 29 shells).

*Partula caledonica* Pfeiffer 1862: Vanuatu: Efate, Teouma Bay, along coast road, L. Price, 23 November 1972 (UF 484595, 4 shells).

*Partula callifera* Pfeiffer 1857: French Polynesia: Society Islands: Raiatea Island, A. Garrett (UF 490681, 1 shell).

*Partula carteriensis* (Quoy & Gaimard 1832): Papua New Guinea: New Ireland Province: St. Matthias Island, W. J. Eyerdam (UF 185667, 5 shells).

*Partula citrina* Pease 1866: French Polynesia: Society Islands: Raiatea Island, A. Garrett (UF 111915, 4 shells).

*Partula crassilabris* Pease 1866: French Polynesia: Society Islands: Raiatea Island, W. J. Clench (UF 192733, 34 shells).

*Partula dentifera* Pfeiffer 1853: French Polynesia: Society Islands: Raiatea Island, W. J. Clench (UF 111914, 8 shells).

*Partula faba* Martyn 1784: French Polynesia: Society Islands: Raiatea Island, A. Garrett (UF 490636, 15 shells).

*Partula flexuosa* Hartman 1885: Solomon Islands: New Georgia Islands, Gizo Island (UF 410366, 1 shell).

*Partula formosa* Garrett 1884: French Polynesia: Society Islands: Raiatea Island, Fatimu (UF 111935, 11 shells).

*Partula fusca* Pease 1866: French Polynesia: Society Islands: Raiatea Island, western Vaioara Valley (UF 111942, 7 shells).

*Partula garretti* Pease 1864: French Polynesia: Society Islands: Raiatea Island: W. H. Pease (UF 111963, 4 shells); (UF 158657, 18 shells).

*Partula gibba* Ferussac 1821: Mariana Islands: Guam Island, W. H. Eshnaur (UF 195878, 7 shells).

*Partula guamensis* Pfeiffer 1846: Federated States of Micronesia: Pohnpei Island, W. H. Pease (UF 192739, 6 shells).

*Partula hebe* Pfeiffer 1846: French Polynesia: Society Islands: Raiatea Island: W. H. Pease (UF 195879, 19 shells); Apoa Valley (UF 111996, 15 shells).

*Partula hyalina* Broderip 1832: French Polynesia: Society Islands: Tahiti Island, A. Garrett (UF 112002, 6 shells).

*Partula imperforata* Pfeiffer 1877: French Polynesia: Society Islands (UF 112013, 4 shells).

*Partula lugubris* Pease 1864: French Polynesia: Society Islands: Tahiti Island, H. J. Armstrong (UF 192734, 18 shells).

*Partula lutea* Lesson 1831: French Polynesia: Society Islands: Bora Bora Island, W. H. Pease (UF 192737, 31 shells).

*Partula macgillivrayi* Pfeiffer 1855: Vanuatu (UF 112033, 6 shells).

*Partula micans* Pfeiffer 1853: Papua New Guinea, P. Dautzenberg (UF 112035, 15 shells).

*Partula mooreana* Hartman 1880: French Polynesia: Society Islands: Moorea Island, A. Garrett (UF 112037, 8 shells).

*Partula navigatoria* Pfeiffer 1850: French Polynesia: Society Islands: Raiatea Island, Western Vaioara Valley (UF 112039, 8 shells).

*Partula otaheitana* (Bruguiere 1789): French Polynesia: Society Islands: Tahiti Island, Faarumai Paune, W. H. Pease (UF 112056, 6 shells).

*Partula planilabra* Pease 1864: French Polynesia: Society Islands: Tahaa Island, A. Garrett (UF 112067, 9 shells).

*Partula radiata* Garrett 1884: French Polynesia: Society Islands: Raiatea Island, A. Garrett (UF 112070, 6 shells).

*Partula radiolata* Pfeiffer 1846: Mariana Islands: Guam Island, N side of Route 7, 5.0 mi E of Route 6, F. Kraus, 17 May 1995 (UF 281058, 20 shells).

*Partula rosea* Broderip 1832: French Polynesia: Society Islands: Huahine Island, A. Garrett (UF 112096, 4 shells).

*Partula rustica* Pease 1866: French Polynesia: Society Islands: Raiatea Island, W. J. Clench (UF 192740, 37 shells).

*Partula similaris* Hartman 1886: Papua New Guinea: Milne Bay Province: Woodlark Island, Guasopa, 9.2241°S, 152.9439°E, J. Slapcinsky, 23 January 2003 (UF 328339, 50 shells).

*Partula suturalis* Pfeiffer 1855: French Polynesia: Society Islands: Moorea Island, A. Garrett (UF 490638, 7 shells).

*Partula taeniata* Mörch 1850: French Polynesia: Society Islands: Moorea Island, A. Garrett (UF 112134, 4 shells).

*Partula thalia* Garrett 1884: French Polynesia: Society Islands: Raiatea Island, W. J. Clench (UF 158680, 16 shells).

*Partula turneri* Pfeiffer 1860: Vanuatu: Anatom Island (UF 112150, 4 shells).

*Partula umbilicata* Pease 1866: Samoa: Upolu Island (UF 192724, 18 shells).

*Partula varia* Broderip 1832: French Polynesia: Society Islands: Huahine Island, T. Dranga (UF 158682, 21 shells).

*Samoana abbreviata* (Mousson 1869): American Samoa: Tutuila Island: T. Dranga (UF 192741, 14 shells); (UF 158646, 8 shells).

*Samoana conica* (Gould 1846): Samoa: Upolu Island, H. J. Armstrong (UF 192732, 14 shells).

*Samoana fragilis* (Ferussac 1821): Northern Mariana Islands: Rota Island, Water Cave, S. Bauman, 2 April 1996 (UF 481329, 1 shell).

*Samoana ganymedes* (Pfeiffer 1846): French Polynesia: Marquesas Islands (UF 195874, 2 shells).

*Samoana inflata* (Reeve 1842): French Polynesia: Marquesas Islands, A. Garrett (UF 490663, 3 shells).

*Samoana strigata* (Reeve 1850): French Polynesia: Marquesas Islands: Nuku Hiva Island, W. H. Pease (UF 192725, 26 shells).
